# Key steps in exposure techniques for robotic total mesorectal excision (TME)

**DOI:** 10.1007/s10151-024-03064-5

**Published:** 2024-12-30

**Authors:** E. P. Tomada, J. Azevedo, L. M. Fernandez, A. Spinelli, A. Parvaiz

**Affiliations:** 1 Department of Biomedical Sciences, Humanitas University, Via Rita Levi Montalcini 4, Pieve Emanuele, 20090 Milan, Italy; 2 IRCCS Humanitas Research Hospital, Via Manzoni 56, Rozzano, 20089 Milan, Italy; 3 Colorectal Surgery, Champalimaud Foundation, Av. Brasilia, 1400-038 Lisbon, Portugal; 4 Faculty of Medicine, University of Lisbon, Av. Prof. Egas Moniz MB, 1649-028 Lisbon, Portugal; 5 Faculty of Science and Health, University of Portsmouth, Winston Churchill Ave, Southsea, Portsmouth, PO1 2UP UK

**Keywords:** Robotic surgery, Rectal cancer, TME, LA, Exposure, Tractions, Modular training

## Abstract

**Aim:**

The use of robotic surgery is increasing significantly. Specific training is fundamental to achieve high quality and better oncological outcomes. This work defines key exposure techniques in robotic total mesorectal excision (TME). Based on a modular approach, macro- and microtractions for exposure in every step of a robotic TME are identified and described. The aim is to develop a step-by-step technical guide of the exposure techniques for a robotic TME.

**Methods:**

Twenty-five videos of robotic rectal resections performed at Champalimaud Foundation (Lisbon, Portugal) with the Da Vinci™ Xi robotic platform were examined. Robotic TME was divided into modules and steps. Modules are essential phases of the procedure. Steps are exposure moments of each module. Tractions are classified as macro- and microtractions. Macrotraction is the grasping of a structure to expose an area of dissection. Microtraction consists in the dynamic grip of tissue to optimize macrotraction in a defined area of dissection.

**Results:**

The procedure videos reviewed showed homogeneity concerning surgical methodology. Eight modules are outlined: abdominal cavity inspection and exposure, approach to and ligation of the inferior mesenteric vessels, medial to lateral dissection of the mesocolon, lateral colon mobilization, splenic flexure takedown, proctectomy with TME, rectal transection, and anastomosis. Each module was divided into steps, with a total of 45 steps for the entire procedure. This manuscript characterizes macrotraction and microtraction fine-tuning, detailing the large-scale macrotractions and the precision of microtractions at each step.

**Conclusion:**

Tissue exposure techniques in robotic TME are key to precise dissection. This modular guide provides a functional system to reproduce this procedure safely; the addition of the exposure techniques could serve as a training method for robotic rectal cancer surgery.

**Supplementary material:**

The online version of this article (10.1007/s10151-024-03064-5) contains supplementary material, which is available to authorized users.

## Introduction

Thanks to its advantages robotic surgery will surpass laparoscopy and open surgery in multiple procedures by 2025 [1] . Total mesorectal excision (TME), introduced by Heald in the mid-1980s [2, 3], remains the gold standard for rectal cancers below the peritoneal reflection. Robotic TME has proved to be comparable to laparoscopy in terms of quality metrics, potentially reducing conversion rates and complications [4, 5]. Colorectal surgeons shifting to robotic platforms need specialized training [6]. A modular system, tested in laparoscopic colorectal surgery in England [7], enables trainees to complete most laparoscopic operations and could facilitate this transition. Several step-by-step guides to robotic TME [8] have been published, focusing on descriptions of the steps in the procedure in chronological order. However, tissue exposure, critical to the performance of any dissection, is often overlooked. In robotic surgery, close vision and three-wristed operating arms emphasize its importance. Proper traction enables exposure of the dissection area, separating avascular spaces of different embryological origin to isolate bowel and mesentery for resections.

This manuscript aims to outline exposure techniques in robotic low anterior resection (LAR) with TME. A modular approach with a description of every step is proposed.

## Materials and methods

Twenty-five videos of robotic anterior resection performed with Da Vinci™ Xi system at Champalimaud Foundation were examined. All patients  signed an informed consent to imaging, video recording, and data publication. Ethical approval was obtained. Each video was assessed by a minimum of two surgeons. The intervention was segmented into modules, then further subdivided into steps. Modules represent major phases of the procedure. Steps are key moments of exposure, marked by changes in traction for dissection. Tractions are pivotal for exposure; they are classified as macro- or microtractions. A macrotraction is the grasping of structures (organ or mesentery) to adequately expose dissection areas. A microtraction is the dynamic grip or exposure of tissue to optimize macrotractions and guide dissection paths.

In multimedia content, robotic first arm instrument tractions are depicted in green, those by the fourth arm in blue, and laparoscopic assistant ones in yellow. Table 1 summarizes the modules.

**Table 1:** Modular division of robotic low anterior resection with TME

Module 1	Abdominal cavity inspection and exposure of surgical field
Module 2	Approach to inferior mesenteric vessels and lymphadenectomy
Module 3	Medial to lateral dissection of left and sigmoid mesocolon
Module 4	Lateral colon mobilization
Module 5	Splenic flexure takedown
Module 6	Proctectomy—TME
Module 7	Rectal transection
Module 8	Anastomosis

### Patient setup and positioning

**Fig. 1:**
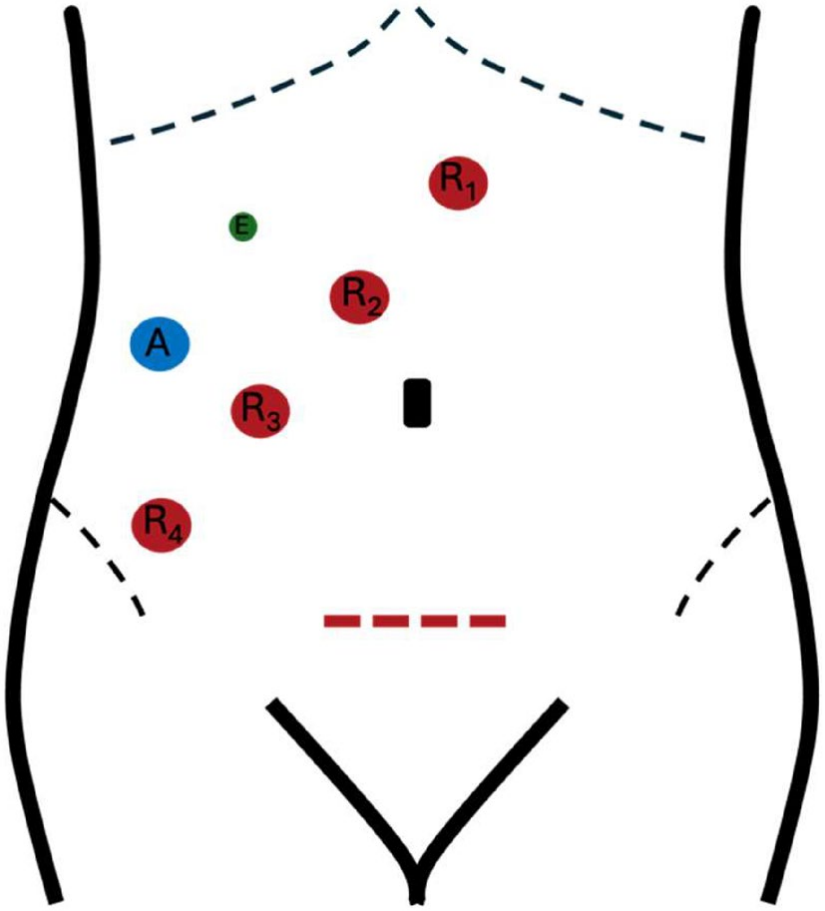
Robotic setup. The Pfannenstiel incision is depicted by a dotted red line, the robotic trocars are drawn as red dots along a diagonal line from the right lower quadrant to the epigastrium with a 6–8 cm distance between one another, the assistant trocar is represented with a blue dot, and an eventual additional trocar is represented in green

The patient is in Lloyd-Davis position, with a Trendelenburg tilt of approximately 20–22° and rotated about 8–10° to the right. A Pfannenstiel incision is made for specimen extraction; four robotic trocars are placed 6–8 cm apart on a diagonal line from the right lower quadrant to the epigastrium on the right of the umbilicus (Fig. 1). An assistant trocar aligns with the third robotic trocar in the right lower quadrant, where AirSeal^®^ is utilized. An additional 5-mm assistant port may be needed for lower tumors and complex cases.

### Module 1—surgical field exposure

**Fig. 2:**
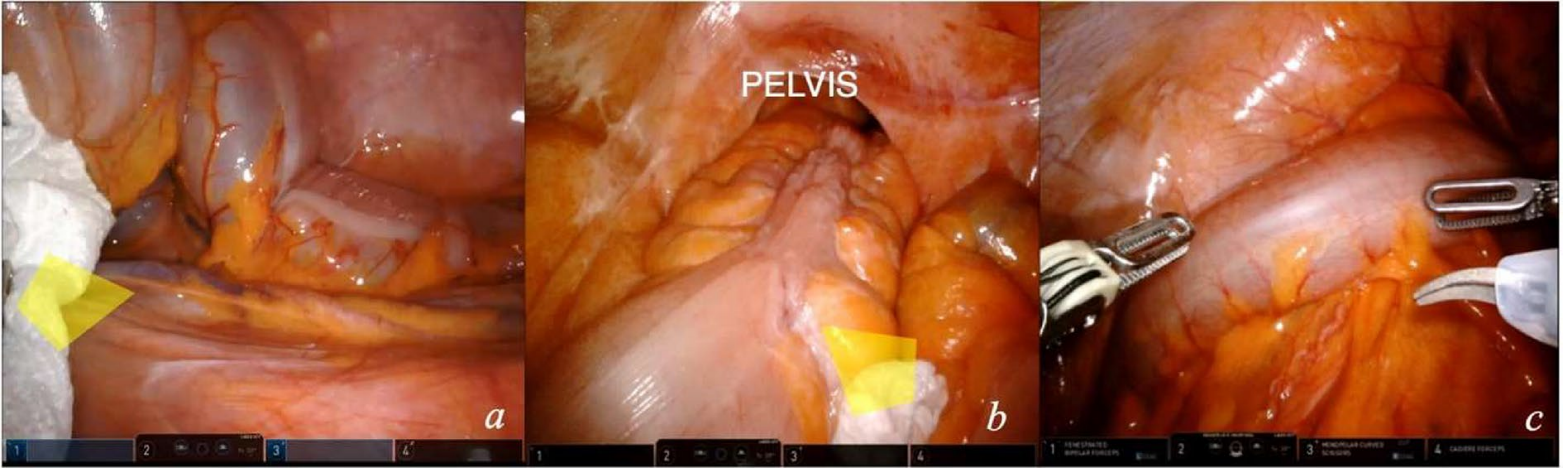
Surgical field exposure. A gauge is used to maintain small bowel loops on the right abdomen, the assistant forceps applies a pressure upward at the level of the ligament of Treitz to expose the mesentery of the left colon (**a**); an additional gauge can be used and placed in the lower right abdomen to keep bowel loops away from the operating area (**b**); docking is performed (**c**)

To start the procedure, exploratory laparoscopy to exclude metastatic spread of the disease is necessary. Exposure of the surgical field (video 1): 

1. Adhesiolysis may be necessary, according to patient conditions and previous surgical history.
2. Greater omentum is gently positioned, without traction, in the left hypochondrium.
3. Small bowel loops are swept, without grasping, to the right abdominal quadrants, firstly near the Treitz, exposing the origin of the inferior mesenteric vessels, secondly to the right lower quadrant.
4. A gauze is placed to maintain the position of the bowel loops at the ligament of Treitz (Fig. 2a).
5. An additional gauze can displace the bowel loops in the lower right abdomen to expose the pelvis (Fig. 2b).

At the end of this module, docking (Fig. 2c) of the robot is performed. The suggested setting for the instruments is the bipolar fenestrated forceps in arm 1, the robotic camera in arm 2, the monopolar curved scissors on arm 3, and the Cadière forceps in arm 4.

### Module 2—approach to the inferior mesenteric vessels and lymphadenectomy

#### Module 2.1—inferior mesenteric artery (IMA) ligation

Starting the procedure with proper exposure is key to initiate dissection in the correct plane. This module is represented in video 1.

**Fig. 3:**
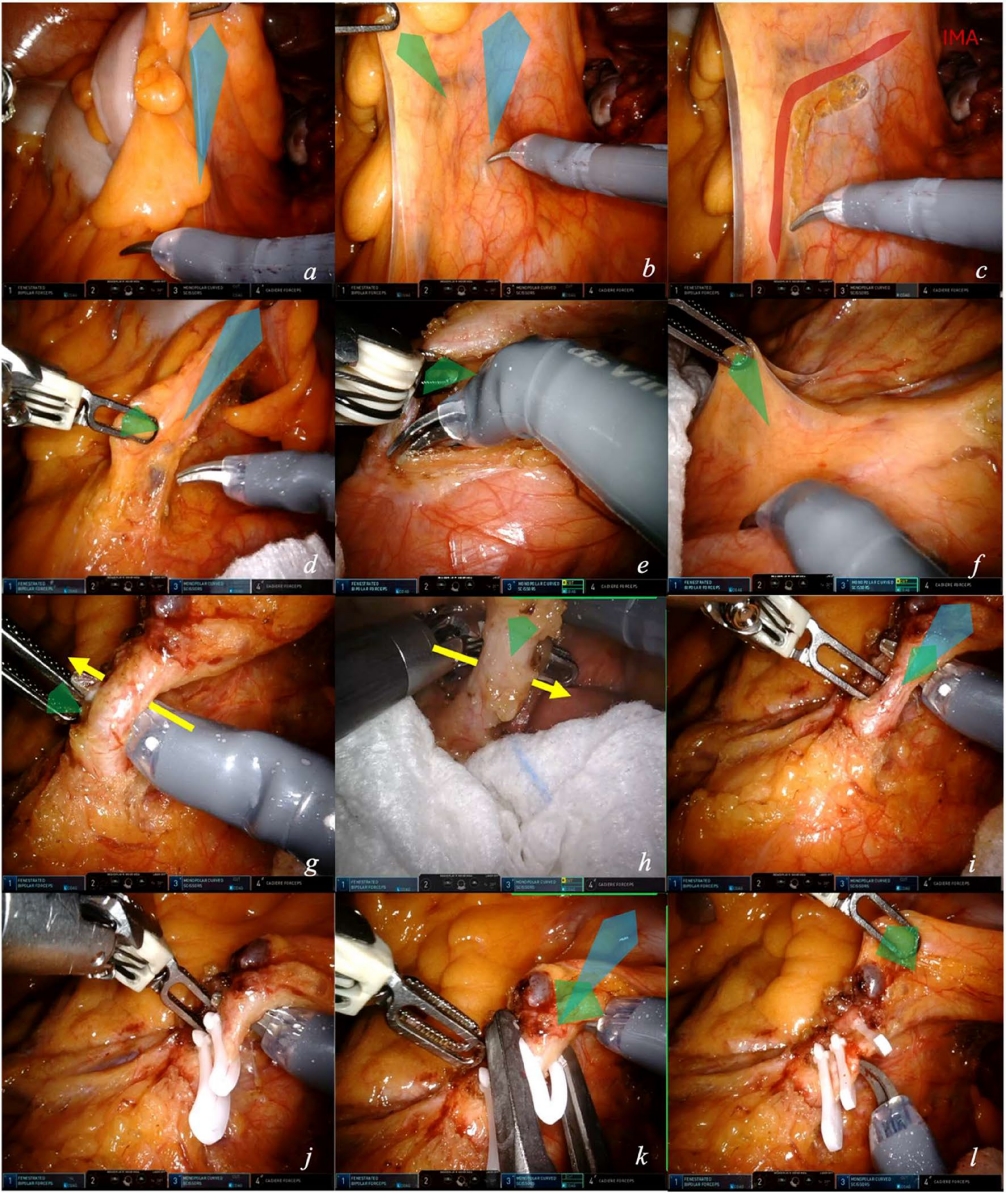
Approach to the IMA. Step by step (**a**–**l**) exposure techniques with depiction of macro- and microtractions for isolation and ligation of IMA

1. Exposure of the mesocolic root is obtained with a macrotraction by arm 4 on the sigmoid colon upward to extend the peritoneum and distance it from retroperitoneal structures (Fig. 3a). An up-and-down movement may highlights the dissection plane with sliding of the retroperitoneal fascia covering the structures.
2. The first arm applies microtraction (Fig. 3b) on the mesentery cranially and medially for exposure of the IMA.
3. The macrotraction and the optimizing microtraction expose the IMA archiform projection on the mesenteric surface (Fig. 3c). Dissection separates the mesocolon from the retroperitoneum.
4. The macrotraction of arm 4 moves proximally on the arch of the IMA to increase tension on the vessel. This allows skeletonization and high ligation of the IMA. A first-arm microtraction makes IMA isolation easier (Fig. 3d).
5. The first arm slides below the artery to facilitate dissection at the origin of the vessel (Fig. 3e). The correct plane of dissection below the artery is identified for retroperitoneal dissection.
6. To obtain complete IMA skeletonization, the window below the inferior mesenteric vein (IMV) can be opened (Fig. 3f). The first arm grasps the peritoneum at the projection of the IMV. The planes of dissection below IMV and IMA are connected around the IMA.
7. The first-arm applies finest microtraction upward and laterally counteracts the force of the scissors to open the space behind the artery at its origin (Fig. 3g). As evident in Fig. 3h, this step can be performed by inverting the forces from the two arms.
8. Once skeletonization is completed, IMA is ready for ligation. The scissors apply a microtraction directly on the artery upward and laterally, parallel to the macrotraction, allowing arterial clipping by the assistant operator (Fig. 3i). A gauze can be positioned posterior to the arterial origin for safe ligation.
9. This double macro- and microtraction at the level of the artery enables adequate high-level ligation, with two locking clips (Hem-o-loks) at 1 cm from aortic origin to avoid damage to sympathetic nerve trunks (Fig. 3j). High ligation guarantees accurate lymphadenectomy.
10. The microtraction of the scissors is displaced more distally to allow for distal clip closure (Fig. 3k). After the clip applier is in place, scissors are removed, and the clip is positioned distally on the vessel.
11. For vessel section, arm 1 microtraction is performed distally to the distal clip (Fig. 3l).

#### Module 2.2—inferior mesenteric vein (IMV) ligation

The ligation of the IMV is crucial in left colon mobilization (video 1). In general, the site of ligation of the IMV is at the level of the ligament of Treitz, below the inferior border of the body of the pancreas.

**Fig. 4:**
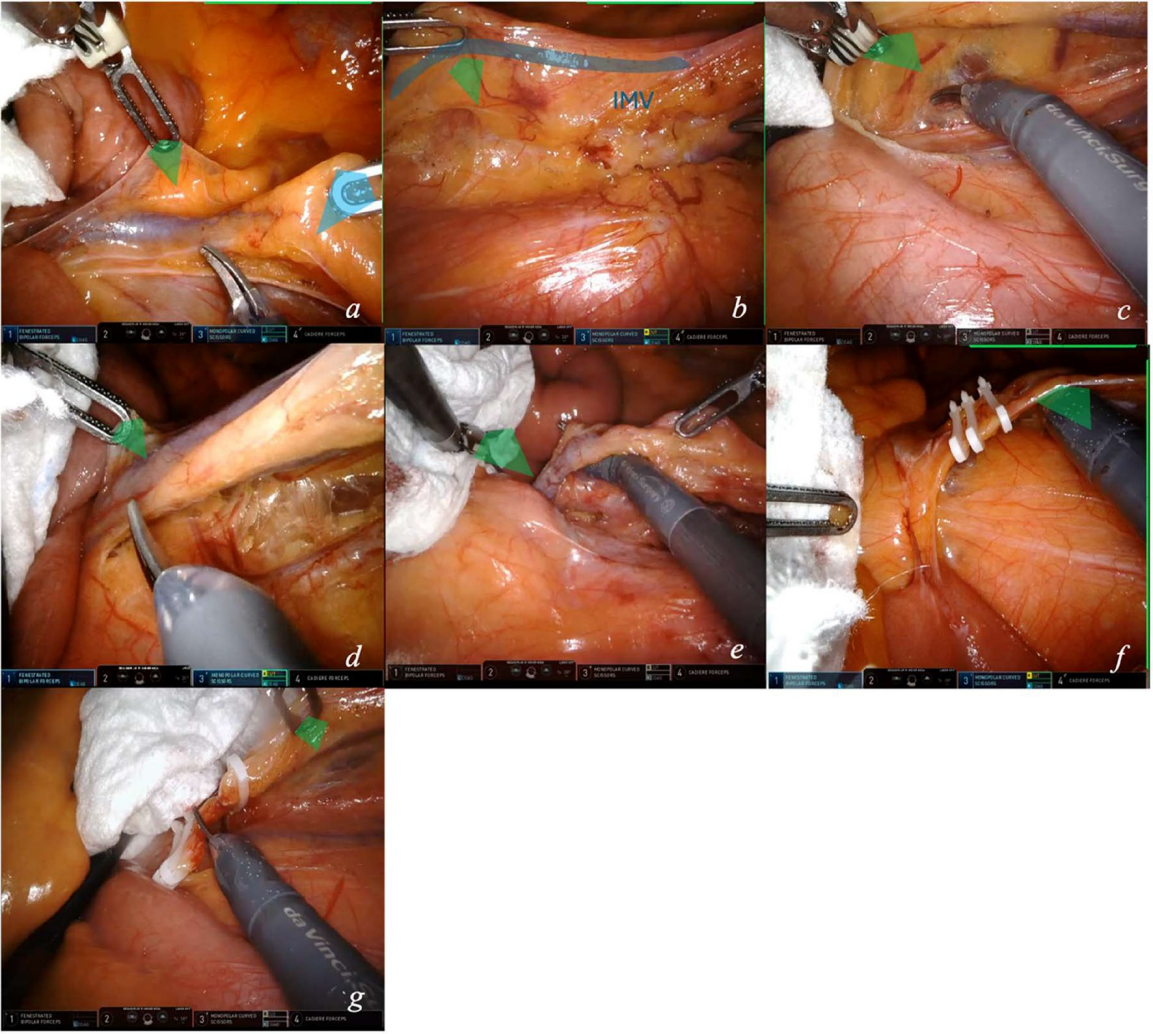
Approach to IMV. Step-by-step (**a**–**g**) exposure techniques with depiction of macro- and microtractions for isolation and ligation of IMV

1. The first arm grasps the peritoneal surface at the IMV (Fig. 4a). This microtraction exposes the vein (Fig. 4b) and the dissectable mesocolic plane. Arm 4 pulls the vein upwards and distally (macrotraction) to increase vein exposure, cranial to the artery.
2. The first arm slides below the vessel, supporting it, and continues to exert traction for innermost mesocolic plane dissection from medial to lateral (Fig. 4c). IMV is isolated and skeletonized.
3. As for IMA isolation, the first arm pulls upward (fine microtraction) for passage of the scissors around the IMV (Fig. 4d, e).
4. The assistant applies a pressure on the gauze for field exposure, facilitating IMV complete visualization and ensuring distance from the left colic vein. To ligate the vessel (either two or one proximal and one distal locking clips), the scissors perform a microtraction, aligned to the fourth arm, sliding below the vessel distally (Fig. 4f).
5. To optimize traction on the vein for section, arm 1 exerts a microtraction distal to the distal clip, or proximal to the clips (Fig. 4g). The vein is sectioned.

### Module 3—medial to lateral dissection of left and sigmoid mesocolon

Once vessels are ligated, medial to lateral dissection on the innermost dissectable plane of the left colon mesentery is performed (video 2).

**Fig. 5:**
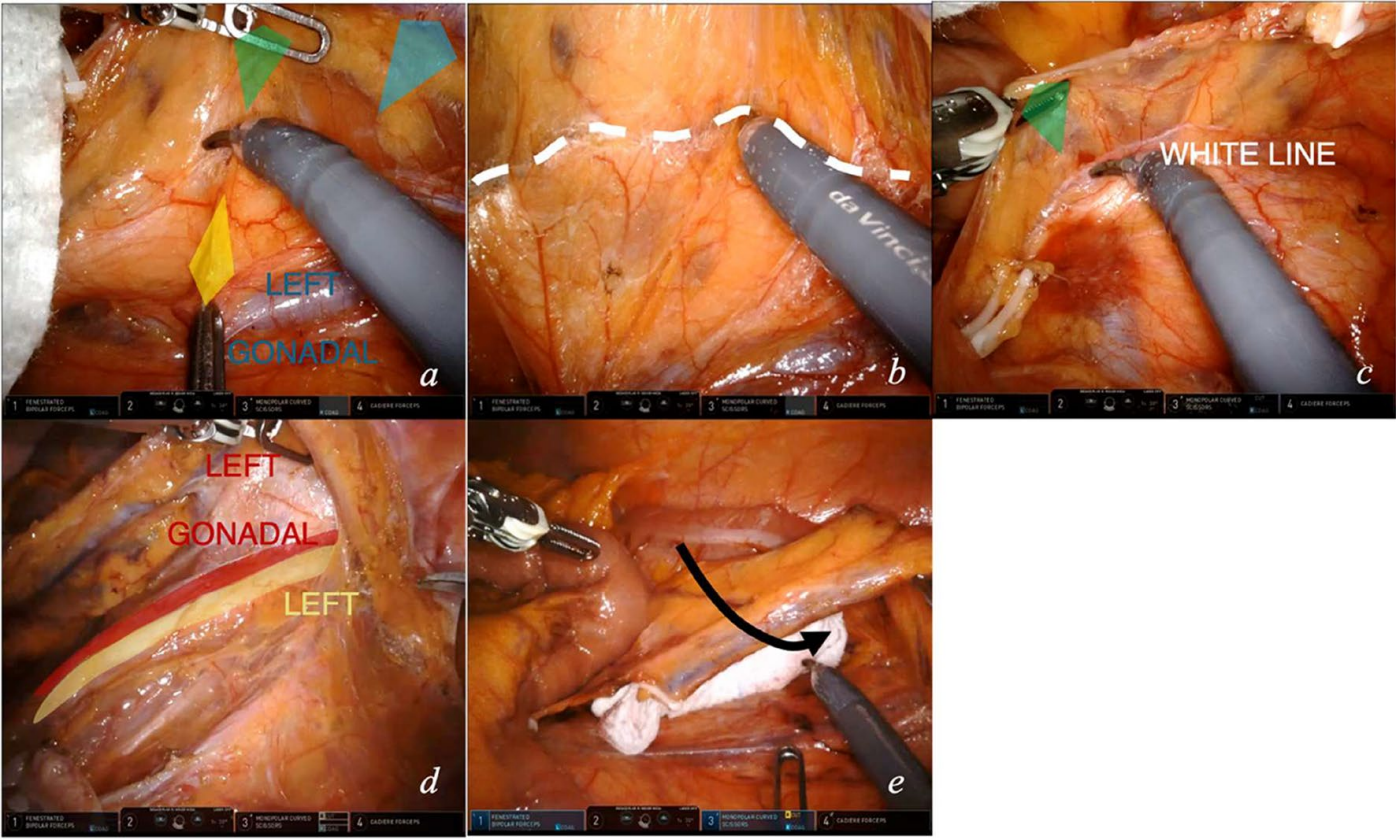
Medial to lateral dissection. Key exposure steps (**a**–**e**) for medial to lateral dissection

1. The fourth arm exerts a macrotraction at the level of the IMV, proximal to the site of IMA ligation, directed caudally and upward; the first arm guides the dissection applying a dynamic microtraction upward, above the dissection plane. The assistant grasps Gerota’s fascia, exerting a counter-microtraction (Fig. 5a, yellow). The innermost dissectable plane is revealed, outlined in white in Fig. 5b. This step is dynamic. Dissection advances while microtractions are displaced laterally. The white line of Toldt is exposed (Fig. 5c). Cranially, the inferior border of the pancreas is dissected from the left mesocolon.
2. Moving caudally, below the site of ligation of the IMA, arm 4 slides below IMA, and arm 1 moves below the mesentery, cranially to the fourth arm. These tractions allow for mesentery tenting. Retroperitoneal dissection is performed by sweeping with scissors on the fascia.
3. The plane of dissection above and below IMA origin are united, and complete mesentery tenting is obtained. Left gonadal vessels and left ureter are preserved (Fig. 5d). A gauze can be placed below the mesentery.
4. Dissection should continue to the sacral promontory, if straightforward from this position.

### Module 4—lateral colon mobilization

At completion of medial to lateral mobilization, when the left colon is visible, lateral dissection is started (video 2). In most of the videos analyzed, lateral dissection follows medial mesentery dissection. Sometimes, lateral dissection is approached first, e.g., very fatty mesenteric planes, mesentery adherent to retroperitoneal structures, previous inflammation of left or sigmoid colon.

**Fig. 6:**
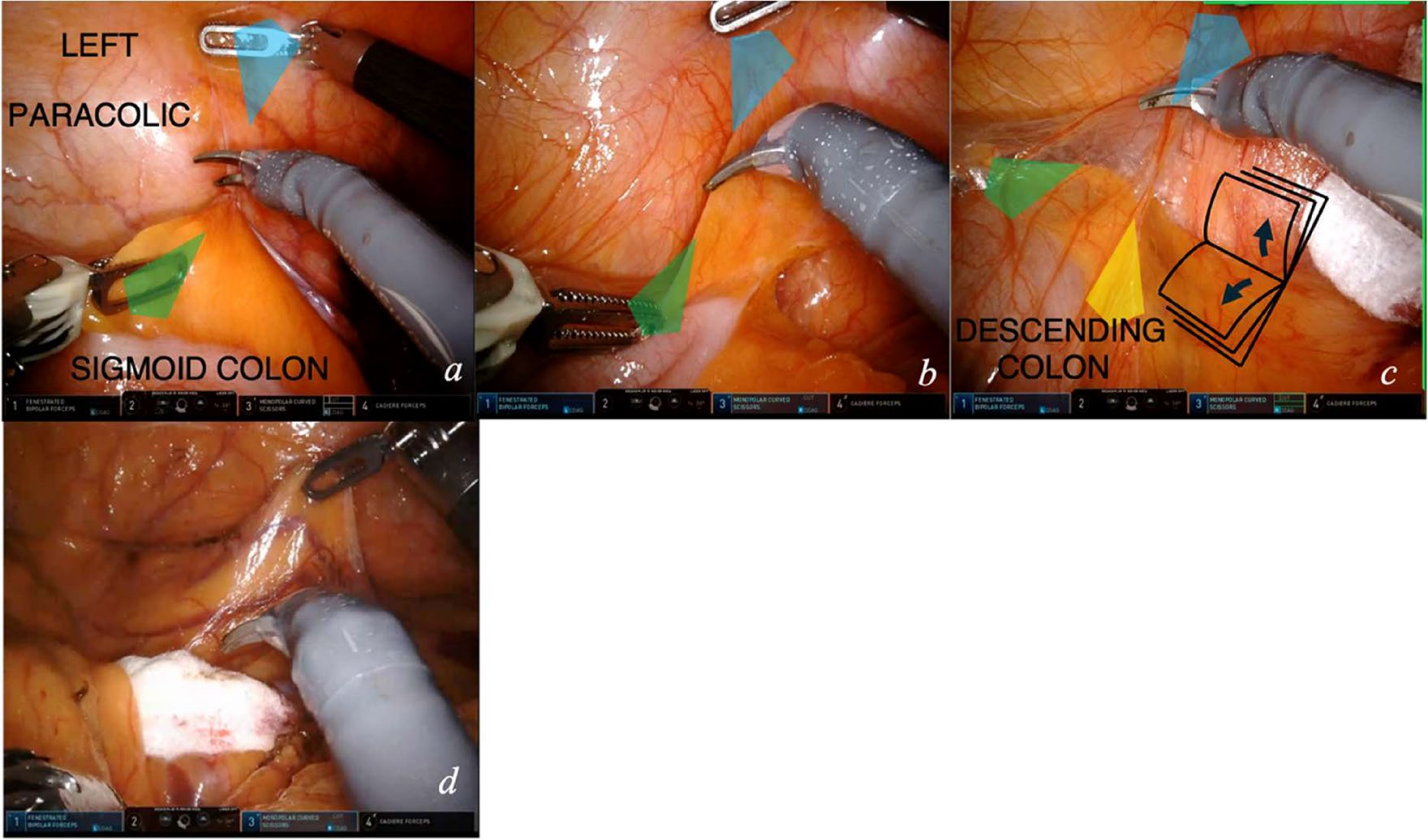
Lateral to medial dissection. Key exposure steps (**a**, **b**) for lateral to medial dissection and connection with the medially dissected plane (**c**)

1. The fourth arm stretches the lateral parietal peritoneum at the passage of the descending to sigmoid colon. Meanwhile, dynamic traction is performed by the first arm on the fat of the left colon, thus the plane of dissection is displayed (Fig. 6a).
2. The dissection of the left colon mesentery from the parietal peritoneum proceeds in a caudal to cranial direction with continuous traction. A methaphorical image to represent the surgeons’ hand movements is that of “opening a book” (Fig. 6b). The two hands perform an internal to external rotation. To optimize tissue exposure, an additional traction can be applied by the assistant on the left colon (Fig. 6c). If a gauze was positioned medially, when lateral dissection reaches the medial, it is displayed (Fig. 6d).

### Module 5—splenic flexure takedown

**Fig. 7:**
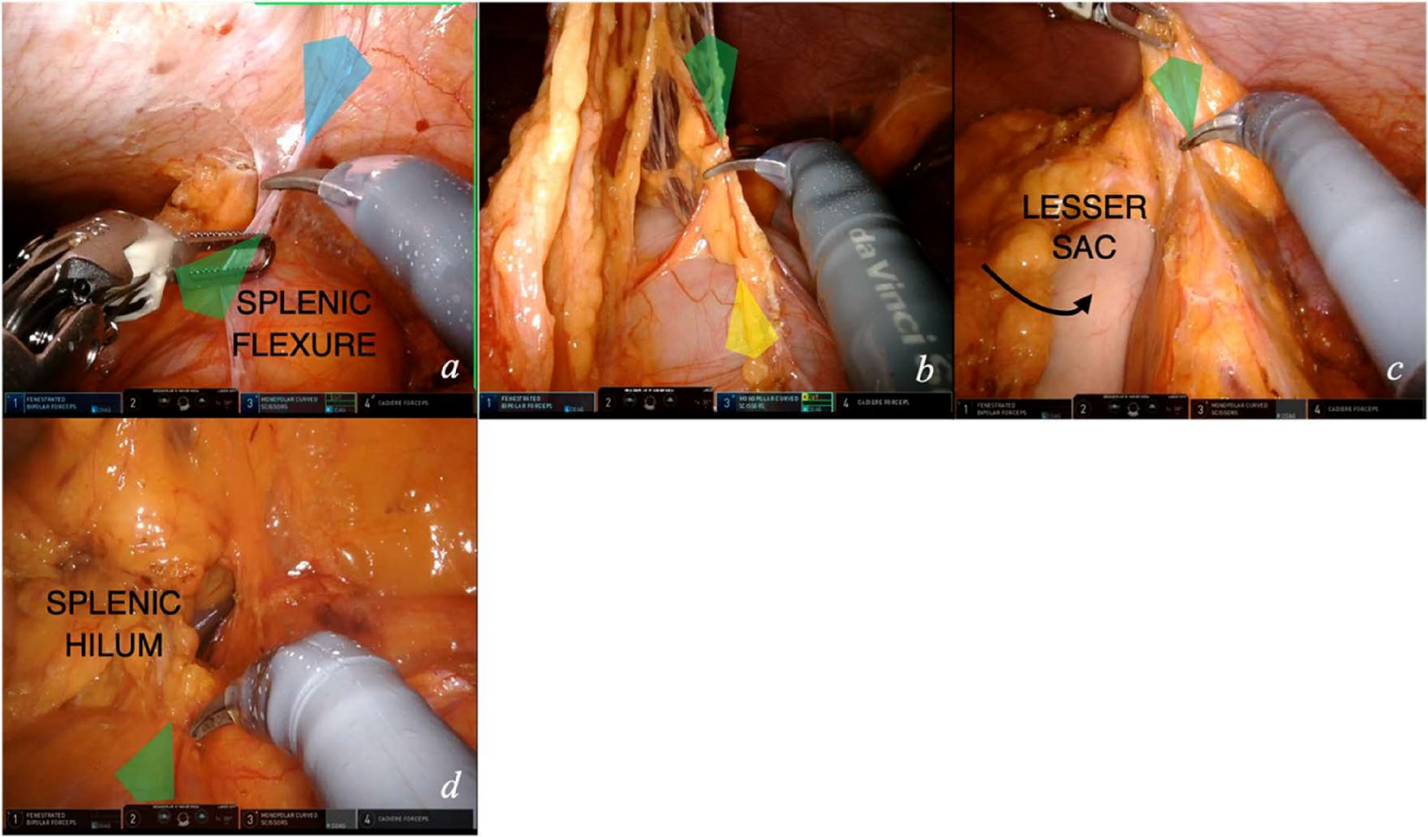
Splenic flexure takedown. Key exposure steps (**a**, **c**) for splenic flexure mobilization, from lateral (**a**) to medial approach with colo-epiploic detachment and opening of the lesser sac (**b**, **c**) and final takedown of the splenic flexure (**d**)

Before starting splenic flexure takedown, robotic arms are rotated, without docking, in a counterclockwise direction to the maximum range. This rotation enables splenic flexure mobilization without arm collision. Two approaches may be performed for splenic flexure takedown. One starts from lateral mobilization and follows the colon until entering the lesser sac. The second approach starts medially, in the transverse colon, and proceeds with the separation of the greater omentum from the colon from medial to lateral before entering the lesser sac. The steps for lateral approach exposure (video 2) can be described as follows:

1. The book-opening maneuver is continued to detach the splenic flexure from lateral attachments (Fig. 7a). The fourth arm is moved to the parietal peritoneum lateral to the splenic flexure. The first arm pulls downward and medially the colon. In this step, the assistant forceps performs an additional traction, equivalent to the one of arm 1 forceps, on the transverse colon.
2. Moving medially, the first arm grasps the greater omentum perpendicular to the transverse colon, the assistant pulls downward the colon; in this way the plane of dissection, close to the bowel wall, is exposed (Fig. 7b). Colo-epiploic detachment is performed. The lesser sac is opened (Fig. 7c). The dissection proceeds laterally connecting with the mesenteric planes dissected laterally and medially.
3. At the level of the attachment to the splenic hilum—or pancreatic tail—traction is performed on the flexure mesentery, gradually dissecting in a centripetal direction (Fig. 7d).

### Module 6—proctectomy with total mesorectal excision (TME)

TME represents the most challenging phase of this intervention (video 3). The ergonomic advantages provided by the robotic platform facilitate it.

**Fig. 8:**
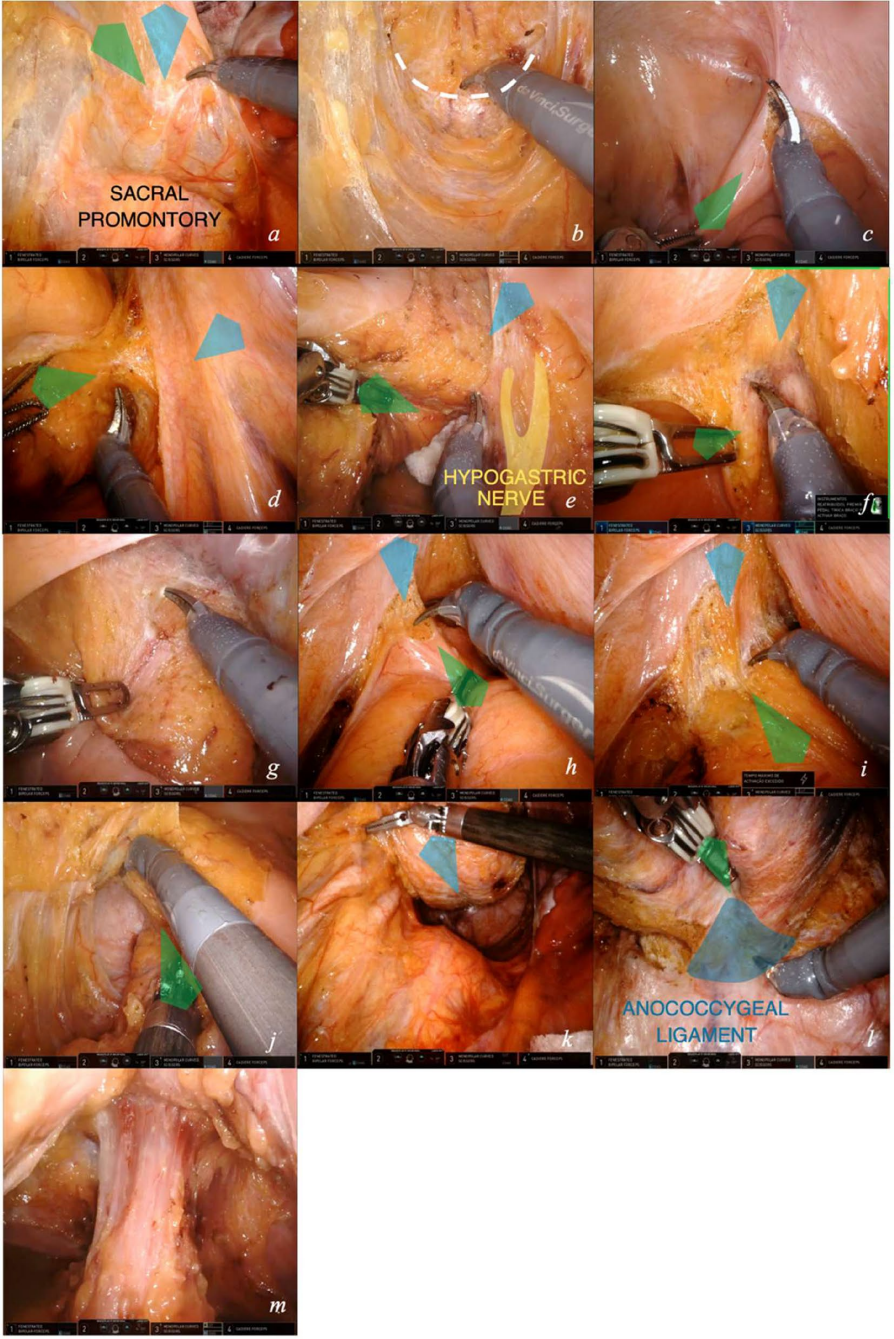
Total mesorectal excision. Key exposure steps (**a**–**m**) for proctectomy with TME

1. The first approach to TME is posterior. The assistant supports the colon, pulling it upward. The fourth arm grasps the sigmoid–rectal junction and extends it upwards–vertically. The first arm slides below the colon to support it. Mesorectal dissection begins posteriorly in between the hypogastric nerves (Fig. 8a). The dissection proceeds downward in the pelvis, centrally, on the innermost dissectable plane. A tunnel is created by the tension of the surrounding tissues (Fig. 8b). The plane of dissection is ventral to the presacral fascia and to Waldeyer’s fascia starting from S2–4. These anatomic landmarks grant safe dissection, avoiding bleeding from presacral veins.
2. When posterior dissection becomes tunneled, lateral dissection is started. The fourth arm releases the colon, arm 1 exerts a microtraction directed rightward on the rectal wall to expose the right border of the peritoneal reflection, which is marked (Fig. 8c). Dissection of the mesorectum continues to the right side and posteriorly, connecting the two planes. To expose the area, arm 4 is positioned on the right pelvic wall, straining the parietal peritoneum (Fig. 8d). The left hypogastric nerve is visualized and carefully preserved (Fig. 8e).
3. Dissection progresses anteriorly, the peritoneal reflection is marked, then mesorectal dissection starts. The fourth arm tractions the anterior parietal peritoneum below the pubis. The first arm pulls the rectal visceral peritoneum downward centrally (Fig. 8f).
4. Anteriorly, Denonvilliers’ fascia [9] is found, a membranous lamina that continues with the peritoneal cul de sac. For non-anterior rectal tumors, Denonvilliers’ fascia is sectioned (Fig. 8g) below the level of seminal vesicles in male; the plane of dissection is located immediately dorsal to it. The first arm microtraction is exerted on the mesorectum downward and centrally.
5. Mesorectum mobilization is obtained when anterior and posterior planes join by left lateral dissection (Fig. 8j). To achieve this, the fourth arm tractions laterally the left parietal peritoneum. First Arm 1 pulls to the right the peritoneal reflection (Fig. 8h) and secondly  to the left mesorectum. The first arm dynamic traction opens the plane of dissection (Fig. 8i). With a slow and continuous increase in traction on the mesorectum, the neurovascular bundle is freed from the mesorectum.
6. When approaching the lower portion of the mesorectum posteriorly, traction on the posterior mesorectum is performed upward by the fourth arm forceps, below the rectosigmoid junction. This can be called the “hockey-stick” maneuver because of the position of the articulated forceps, as seen in Fig. 8k. This traction allows for visualization of the so-called *Bill’s buttocks*, after complete freeing of the mesorectum.
7. Dissection continues distally until it reaches the pelvic floor. The robotic camera is inverted to optimize vision. The first arm applies a microtraction on the posterior mesorectal fascia and exposes the anococcygeal ligament (Fig. 8l) [10]. Careful entry in the pelvis by the instruments is crucial at the pelvic inlet with the sacral promontory.
8. Rectal wall exposure is performed circumferentially at the level defined for section. The first arm retracts the rectal wall for isolation (Fig. 8m).

### Module 7—rectal transection

**Fig. 9:**
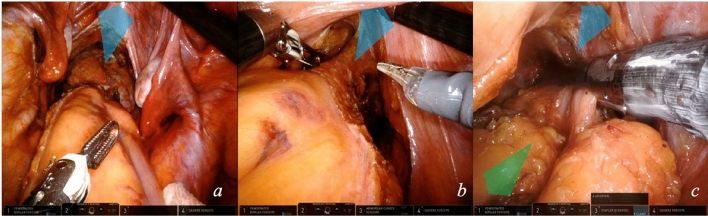
Rectal transection. Exposure of the female pelvis (**a**) and of the male pelvis (**b**). Linear stapling of the distal rectum (**c**)

1. In a female patient, the fourth arm supports the uterus upward; the first arm tractions posteriorly the rectal wall (Fig. 9a) so the robotic stapler is positioned at the level of transection.
2. In a male patient, the Cadière forceps exposes anteriorly the pelvis (Fig. 9b). The instrument may be replaced by a Tip-Up grasper when extra exposure in a deep pelvis is needed.
3. Rectal transection is completed by stapler firing. The fourth arm widens the pelvis by tractioning the peritoneal fold anteriorly; arm 1 calibrates the direction of the rectum for stapling. Firing is performed in an anterior to posterior direction (Fig. 9c). More than two firings should be avoided.

This module is displayed in video 3.

### Module 8—anastomosis

After stapling, the mesentery is sectioned and bowel perfusion can be checked by real-time angiography; these steps can be performed extracorporeally. Proximal section of the colon is performed extracorporeally. The anvil is secured, and the colon is repositioned intracorporeally. Anastomosis is performed (video 3).

**Fig. 10:**
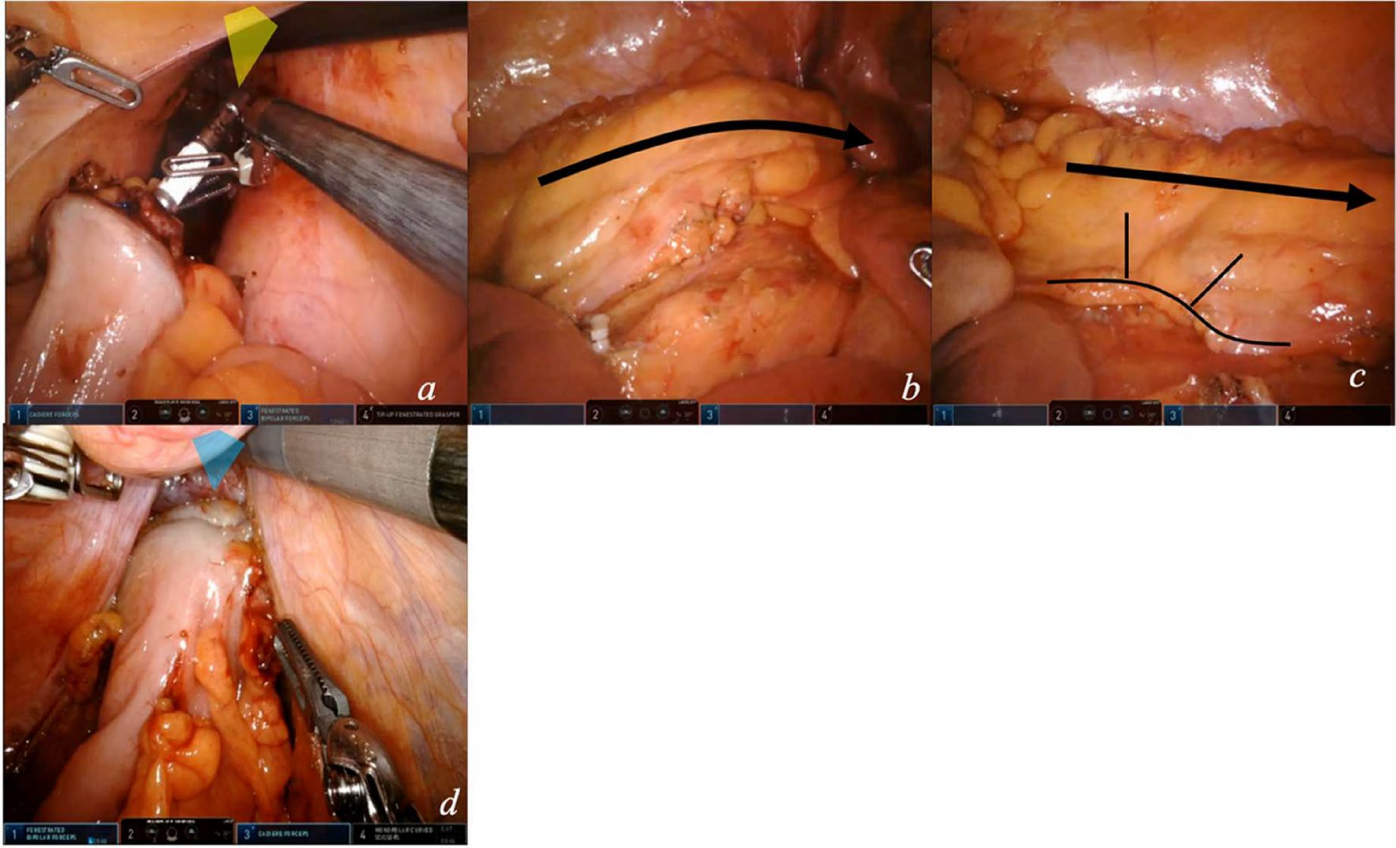
Anastomosis. Colon positioning in the pelvis (**a**), correct mesentery orientation checking (**b**, **c**), and anastomosis construction (**e**)

1. The colon is brought to the pelvis.
2. For the anastomosis, the fourth arm grasps the stapler anvil (Fig. 10a). The assistant, or arm 4, exposes the pelvis by applying a macrotraction anteriorly on the parietal peritoneum.
3. Checking for proper mesentery orientation is fundamental before anastomosis sealing (Fig. 10b, c).
4. Circular end-to-end stapled tension-free anastomosis is performed (Fig. 9d).

## Discussion

Robotic surgery for rectal cancer is becoming the best tool in the hands of dedicated surgeons, offering precision and dexterity surpassing laparoscopy. Literature exists detailing the procedure steps [8]. Tissue exposure, crucial at a practical level, remains unaddressed. Tissue exposure reveals dissection planes and facilitates safe surgical resection. Robotic TME involves several phases in which tissue exposure can be extremely demanding. This work provides a meticulously crafted guide to complete the procedure with adequate exposure. To perform a correct and safe oncological TME exposing the innermost dissectable plane, Heald’s holy plane [11] is key to sharp dissection.

In this work, we sought to convey the essence of a methodology allowing its reproducibility. The high homogeneity in the steps defines how a standardized method is necessary to perform radical surgery in different types of patients (e.g., sex differences, tumor extension, body mass index). Dividing each module into steps according to the direction and amount of traction applied allows for in-depth understanding of the surgery. The multimedia content used to complement the text is unique in supporting the reader.

The main limitation of this work is that videos were collected at a single center. The analysis of a single-center experience guarantees standardization but precludes comparison with different institutions. A minor limitation is the presentation of a single approach to splenic flexure takedown and a single anastomotic configuration.

## Conclusions

A modular approach for exposure techniques in robotic TME is feasible, simple, and reproducible. This step-by-step guide favors robotic TME standardization. This rigorous modular approach enhances oncological safety and the performance of adequate TME. As a future perspective, this methodology has great potential for application in a training program with learning assessment.

## Electronic supplementary material

Below is the link to the electronic supplementary material.

Supplementary material 1 (MP4 198,106 kb)

Supplementary material 2 (MP4 303,133 kb)

Supplementary material 3 (MP4 383,731 kb)

## Data Availability

No datasets were generated or analyzed during the current study.
